# Multiclass Mask Classification with a New Convolutional Neural Model and Its Real-Time Implementation

**DOI:** 10.3390/life13020368

**Published:** 2023-01-29

**Authors:** Alexis Campos, Patricia Melin, Daniela Sánchez

**Affiliations:** Tijuana Institute of Technology, TecNM, Tijuana 22379, Mexico

**Keywords:** real-time, face mask classification, convolutional neural network, COVID-19, computer vision

## Abstract

The world has been greatly affected by the COVID-19 pandemic, causing people to remain isolated and decreasing the interaction between people. Accordingly, various measures have been taken to continue with a new normal way of life, which is why there is a need to implement the use of technologies and systems to decrease the spread of the virus. This research proposes a real-time system to identify the region of the face using preprocessing techniques and then classify the people who are using the mask, through a new convolutional neural network (CNN) model. The approach considers three different classes, assigning a different color to identify the corresponding class: green for persons using the mask correctly, yellow when used incorrectly, and red when people do not have a mask. This study validates that CNN models can be very effective in carrying out these types of tasks, identifying faces, and classifying them according to the class. The real-time system is developed using a Raspberry Pi 4, which can be used for the monitoring and alarm of humans who do not use the mask. This study mainly benefits society by decreasing the spread of the virus between people. The proposed model achieves 99.69% accuracy with the MaskedFace-Net dataset, which is very good when compared to other works in the current literature.

## 1. Introduction

The contingency of the COVID-19 virus has caused people around the world to use face masks as a measure to stop the spread of this disease and in turn, any other disease that can be air transmitted or by contact with other people. Some symptoms that could occur are dry cough, tiredness, fever, and headaches, among many other symptoms that have been occurring and changing throughout the pandemic [1]. Similarly, there are cases in which no symptoms occur. Therefore, it is imperative to stop the spread of infections and prevent humans from catching diseases. People who have been infected have a varied recovery time that could get worse depending on the person who is sick. On many occasions, it is determined that the sick person fulfills quarantine depending on the severity. Facial recognition is a method that has been growing in recent years and the industry has been revolutionizing it through artificial intelligence (AI) and machine learning (ML) techniques. People today relate heavily to facial recognition and using computer vision techniques and image processing makes it possible to solve this complex task. Face masks have become an item of daily use, and, even though the obligation to wear them has been diminished, there are many institutions where it is still a duty to use them; some of these places can range from public locations such as schools, universities to private locations, such as offices. A mask is placed incorrectly when in the human it is not covering the region of the mouth, nose, and chin. Companies and institutes should locate humans who are using the mask correctly or are not wearing it, in a specific area. A real-time system would solve this problem, allowing to identify people who wear a mask, who are using it incorrectly, or who are not wearing one at all, to be identified. The use of convolution neural networks (CNNs) facilitates the identification and classification of images, extracting the main features and locating patterns that can be observed. The objective of our work is to solve this problem through CNN and machine learning techniques to distinguish the appropriate use of face masks.

This article proposes a real-time system using deep learning techniques and computer vision, to classify people into three classes, if they wear masks correctly, if they use masks incorrectly, or if they do not wear masks. The system is carried out using a video camera and a Raspberry Pi 4 combining the use of libraries such as OpenCV and TensorFlow, as well as Python as a programming language. The method will identify a person’s face and place a rectangular box labeled “Mask” if the person is appropriately using a mask, which occurs when a mask covers the nose, mouth, and chin; otherwise, if the person is only wearing a mask on their chin and mouth, the model will label the box as “Incorrect”, and if they are not wearing any mask at all or if it is only on their chin, then the label will be “NoMask”. Therefore, the proposed method will allow the virus transmission to slow down, potentially benefiting people’s health systems.

The dataset used to test the effectiveness of the CNN architecture is MaskedFace-Net. This dataset is provided by Cabani [2] and Hammoudi [3], features around 137,016 images and is based on another Flick-dataset [4]. The MaskedFace-Net images have a size of 1024 × 1024 pixels, and images are internally classified into two subcategories named Correctly Masked Face Dataset (CMFD) and Incorrectly Masked Face Dataset (IMFD).

The Raspberry Pi is a small, low-cost device that can be used as a computer and runs programs in Python [5]. The graphics processing unit (GPU) outputs and inputs all work on a circuit. GPIO Board pins are an important element that allows the RPi to access hardware programming to control the I/O device’s electronic circuitry and data processing. We can add a keyboard, power supply, monitor, and mouse that run on the Raspberry Pi through the HDMI connector. New models are available that can communicate with the Internet via Wi-Fi and Ethernet ports. The RPi can be used with the Raspbian operating system [6].

**Figure 1:**
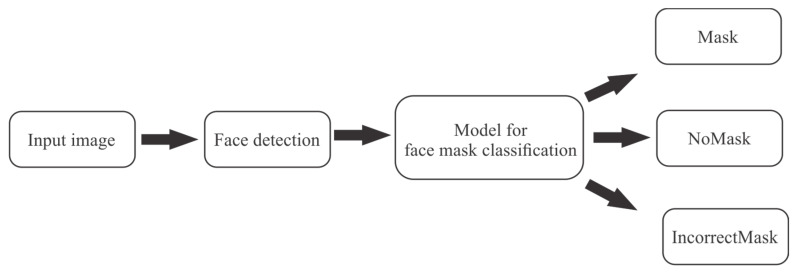
Proposed method to detect the use of face masks.

In summary, this study proposes a CNN model where computer vision, machine learning, and deep learning techniques are combined to achieve classification in the three classes (NoMask, Mask, IncorrectMask). In Figure 1 can be observed that the region of interest is identified by the input image, in this case, the face will be identified and when the image is identified, a CNN model will be used to determine the use of masks to classify it as one of the three categories.

This article is structured as follows, in Section 2 there is information from different authors who investigated the use of face masks, comparing some of their results, followed by Section 3, which deals with the methodology implemented to create the convolutional neural network model, as well as the dataset and preprocessing. In Section 4 the results obtained from the model are presented as real-time examples. Section 5 shows the conclusions, as well as future work. 

## 2. Background

Artificial intelligence and deep learning (DL) are technologies that have been constantly growing and many applications around them have been developed and implemented in the industry, as they have been applied in various areas, such as pattern recognition, and image processing, among others. CNNs have proven to be quite efficient at solving pattern problems, some standard architectures such as Resnet [7], YOLO [8], and MobileNet [9] already have an integrated convolutional neural network model. 

Our research group has worked with convolutional neural networks with diverse goals, as we can find in [10], where a new CNN model in combination with image preprocessing and optimization algorithms was proposed for diabetic retinopathy classification. Additionally, in [11] they use a deep neural network model for guitar classification, including fuzzy edge detection to improve the accuracy. In [12] a new hybrid approach was proposed, using fuzzy logic integration in combination with a modular artificial neural network.

In other works, such as in [13], they propose the use of the MobileNetV2 [14] neural network architecture in combination with single shot detector (SSD) [15] performing real-time prediction using libraries such as OpenCV with an alert system to detect people who use or do not use the mask with the use of a Raspberry Pi 4 achieving between 85% and 95% accuracy percentage. A model called Facemasknet is proposed in [16] where they achieve 98.6% to identify people who are wearing the mask, those wearing improperly, and those who do not have masks. In [17], the authors use a deep learning model to classify images into whether they use masks or no masks, using a small database of 460 images for non-masks and 380 for face masks implementing MobileNetV2. Additionally, in [18], a CNN is utilized to classify humans who are using the mask correctly, incorrectly, or not wearing masks, with the help of the Flickr-Faces-HQ and MaskedFaceNet database, achieving 98.5% accuracy. Haar Cascades is widely used to identify the region of the face as it [19] uses it, in turn with the MobileNetV2 architecture and with the Real Facemask dataset and MaskedFaceNet dataset reaches 99% accuracy.

Similarly, in [20], they use technologies for mask identification, analyzing in real time the category to which it belongs, classifying into two classes Mask and NoMask, adding methods to improve the dataset, and eliminating images with low light.

Several works have managed to detect the use of face masks, and each of them uses a different method to classify the correct use of the masks. It is very common to utilize neural network architectures already tested, for example, MobileNet [21], YOLO [22,23,24], Inception [25] or Resnet [26,27,28] each one reaching different percentages of precision and different type of classification. Other authors [29,30,31], have managed to solve the same problem with their own convolutional neural network models.

Table 1 compares different studies carried out by various authors, where the purpose was to classify the correct use of face masks, generally performing a type of multiclass classification, although many others are based on binary studies.

Most of the authors’ works use Python as a programming language in conjunction with its libraries, such as Tensorflow, OpenCV, and Keras, achieving between 90% and 99% accuracy. Other works have mostly used CNN architectures such as YOLO, MobileNet, or Resnet, whereas we proposed a CNN with one less convolutional layer that is fast to train on the MaskedFaceNet dataset, as we can realize from Table 1, where they proposed different classification models.

Some other related works use the MobileNet architecture to demonstrate that ROpenPose runs faster than a number of the current state-of-the-art models and performs detection similarly [35]. The authors in [36], in order to breakdown and reconstruct spherical iris signals and extract more robust geometric properties of the iris surface, suggest using a spherical–orthogonal–symmetric Haar wavelet. Additionally, in [37], they propose a multi-stage unsupervised stereo matching method based on the cascaded Siamese network. In [38], a two-stage multi-view stereo network is suggested for quick and precise depth estimation.

## 3. Methodology

The proposed real-time system mainly helps to decrease the spread of any infection that is transmitted by air, by identifying people according to the use of face masks. This can be carried out through monitoring and using alarms according to the rules that each location has. This section will cover the proposed solution to solve this problem, as well as the proposed architecture to detect people according to the use of face masks and enclose the region of the face in a rectangle.

**Figure 2:**
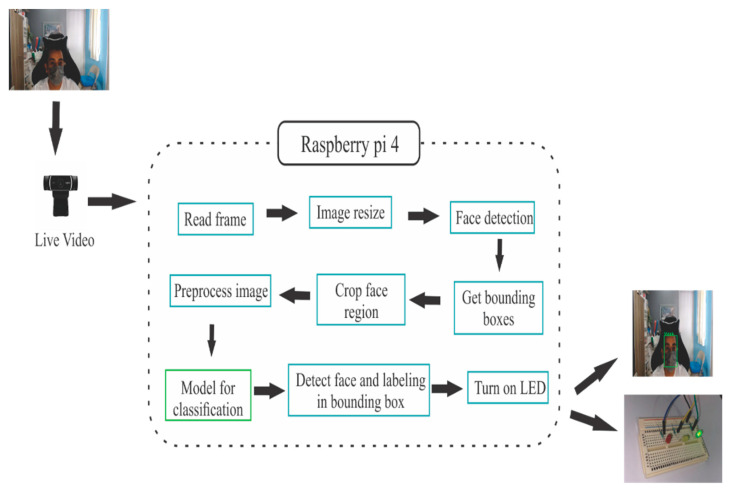
Proposed real-time system architecture.

Basically, the system monitors and identifies the use of masks, see Figure 2, where from our convolutional neural network model it classifies people with real-time images into three classes: Mask which is when the person has a mask and green mark, IncorrectMask when the person has the mask incorrectly identified by the yellow color, and NoMask which is when the human is not using any mask and is indicated by red color. 

The proposed system utilizes computer vision techniques and deep learning techniques to detect the region of the face. There will be an automatic indication, using a Raspberry Pi 4 and a camera, of the people who are using masks, who do not wear masks, or use masks incorrectly.

### 3.1. Convolutional Neural Network

In order to handle challenging image-driven pattern recognition problems, CNNs are typically utilized, and their precise and straightforward architecture makes using artificial neural networks (ANNs) easier, as they are very effective in accessing the graphic properties of the image.

**Figure 3:**
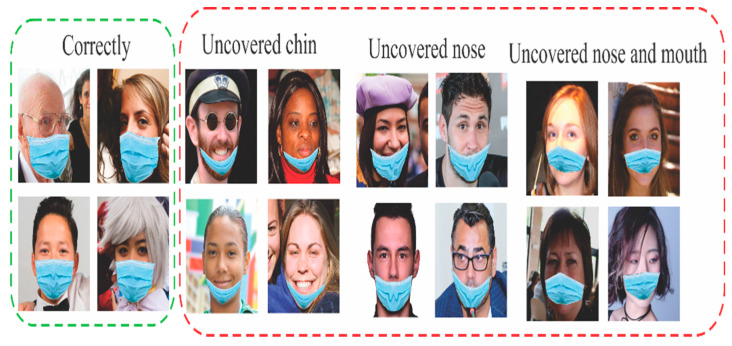
Example of MaskedFace-Net database.

In this case, only MaskedFace-Net images were used, see Figure 3, using the four classes provided by this dataset. Multiple experiments were performed, where each was run 30 times to obtain the average precision and standard deviation, and the best case was identified for each experiment.

The MaskedFace-Net dataset is classified into four classes: correctly masked, uncovered chin, uncovered nose, and uncovered nose and mouth, where the last three are part of a higher class called incorrectly masked. There have been proposed three different models to identify the CNN architecture that offers the best results.

**Figure 4:**
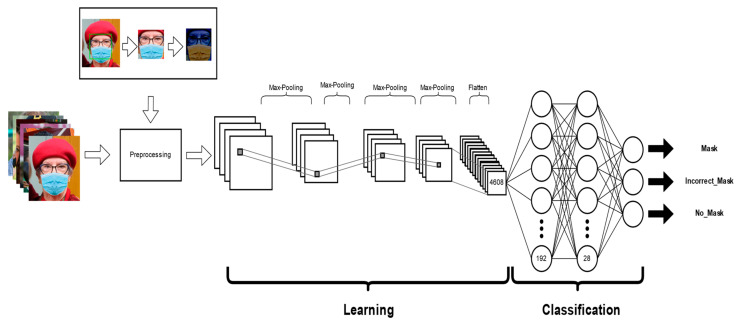
Proposed CNN model architecture.

The architecture of the proposed CNN model basically comprises two stages, where the learning stage contains four convolutional layers with ReLu as the activation function and max pooling applied between each layer, and in the classification stage, the class to which it belongs is identified according to the three proposed classes: Mask, NoMask, and IncorrectMask. Therefore, the proposed general architecture is shown in Figure 4.

This model is designed to classify the correct use of the face masks, utilizing images of the database and applying preprocessing to each of them. Basically, we first find the main characteristics of the region of the face found in the image, then this model in the learning stage uses four convolutional layers applying max pooling between each of them, adding ReLu as activation function. Finally, in the classification stage, it will be assigned to the class that belongs, including Mask, NoMask, or IncorrectMask. We compared other convolutional neural networks models, and this one provided us with better results and great performance.

**Figure 5:**
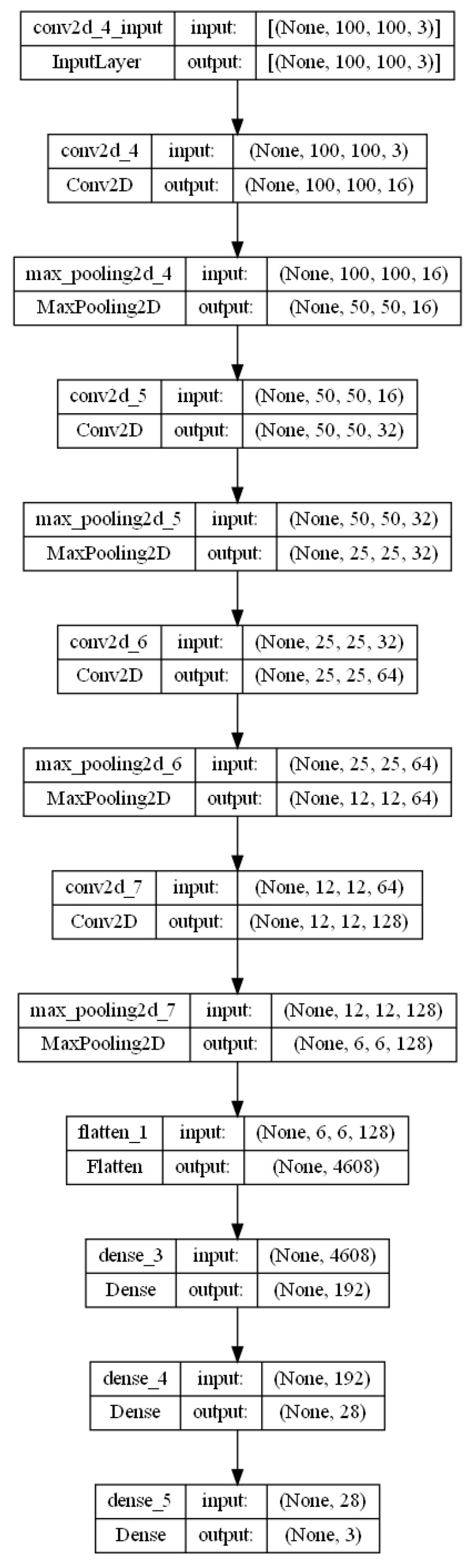
Global model for face mask classification.

As we can note in Figure 5, the global model used to implement our method shows all the convolutional layers, the max pooling as pooling operation and classification stage, ending with a dense layer classified into three classes. 

### 3.2. Database

**Figure 6:**
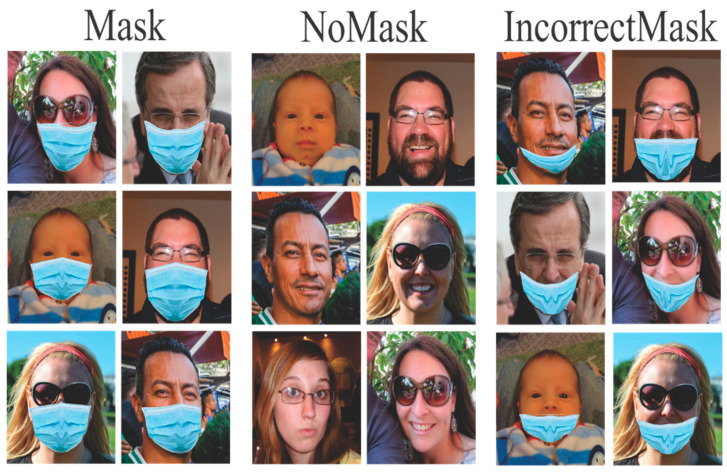
Example of the proposed database.

In this work, three datasets are used to perform the training and testing of the CNN model, one for each class. To create the Mask class, we use the Correctly Masked Face Dataset, also to create IncorrectMask class we utilize the Incorrectly Masked Face Dataset. These two datasets are part of MaskedFaceNet, this dataset has around 137,016 images of faces with simulated face masks, and is based on another dataset, and as mentioned by the authors in [2,3], the images are completely free to use under a license, and the classification efficiency of face masks has been corroborated through test with CNN models. The third dataset is the Flickr-Faces-HQ dataset, which contains images originally 1024 × 1024 in size with a wide variety of people in terms of background, age, or ethnicity, and this dataset was used to create the NoMask class, an example of the content of this dataset can be found in Figure 6.

In addition, the first 15,000 images in total were selected for training, testing, and validation; therefore, 5000 images were used for each class. The CNN training was divided into different percentages where 70% was used for training, 20% was used for testing and the remaining 10% was used for validation. 

### 3.3. Data Pre-Processing

**Figure 7:**
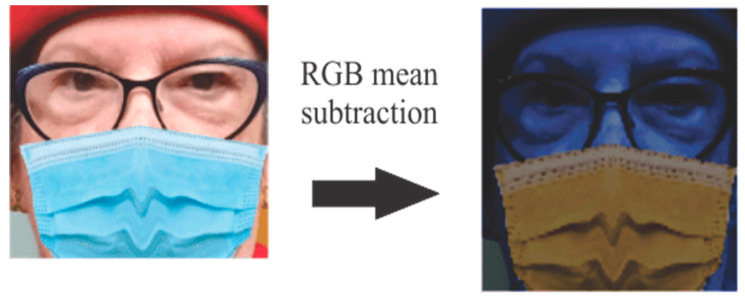
RGB mean subtraction sample.

Image preprocessing using images from the MaskedFace-Net database is performed by classifying and tagging them into three different types of mask wear. To improve the percentage of accuracy, a face detection model well known as the Caffe model [39] was used. For the preprocessing of the image an existing background subtraction was used, in this processing algorithm a technique known as RGB mean [40] subtraction is used, see Figure 7.

This face detection and the preprocessing algorithm are applied to all images of the dataset to facilitate the identification of the mask and the class to which it belongs.

#### Caffe Model

**Figure 8:**
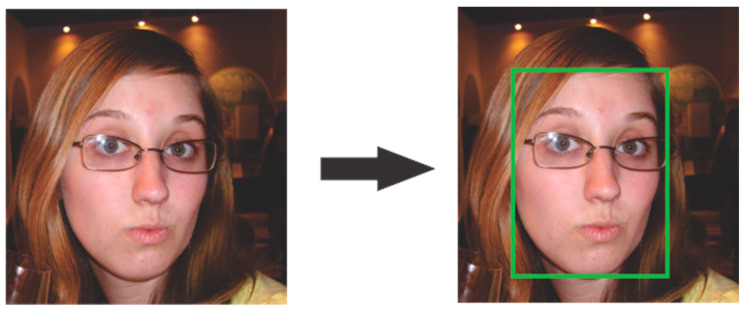
Caffe model face recognition example.

Caffe model is a pre-trained model for the face detection algorithm that uses deep learning and computer vision techniques. This model is very efficient with faces at different angles, it is written in C ++ and provides tools for Python and Matlab. The model for face detection is pre-trained with 300 × 300 images, an example of face detection is shown in Figure 8.

The Caffe model uses the Resnet-10 architecture and is based on Single Shot Multibox Detector (SSD). It is efficient with rapid head movements and occlusion managing to identify the region of the face very well from different sides or angles even when wearing a face mask.

### 3.4. Classification

**Figure 9:**
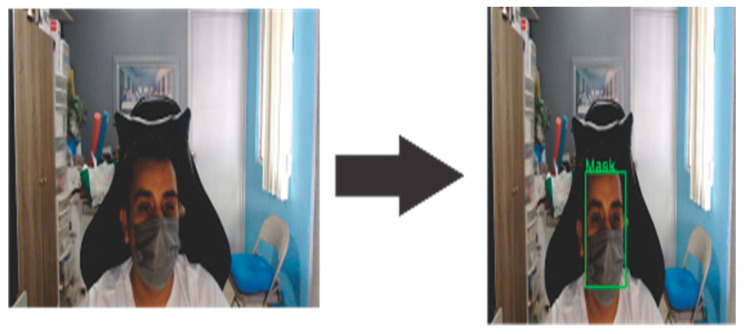
Example of classification of the use of face masks.

Once the face detection was performed and preprocessing applied, the model that has been trained to identify the mask is used to classify the corresponding class, see Figure 9. 

This trained model recognizes the state of the face mask use according to the image obtained from a video camera, recognizing the face region, and was tested with black, gray, and blue face masks. Loading the model enable obtaining a good percentage of accuracy and acceptable results for real-time prediction, Python libraries are basically used for the creation of the model sequence and classification.

### 3.5. Raspberry Pi

A Raspberry Pi 4 is a small device with a 4 GB ARM processor and HDMI inputs, USB, and microSD ports. The operating system is based on GNU/Linux and is called Raspberry Pi OS, which is a custom version of Debian. The proposed system uses it in combination with a camera to obtain the image in real-time, to monitor and identify the use of face masks. Our system basically performs face detection through the model loaded on the Raspberry Pi, in addition to identifying the real-time status of mask use.

**Figure 10:**
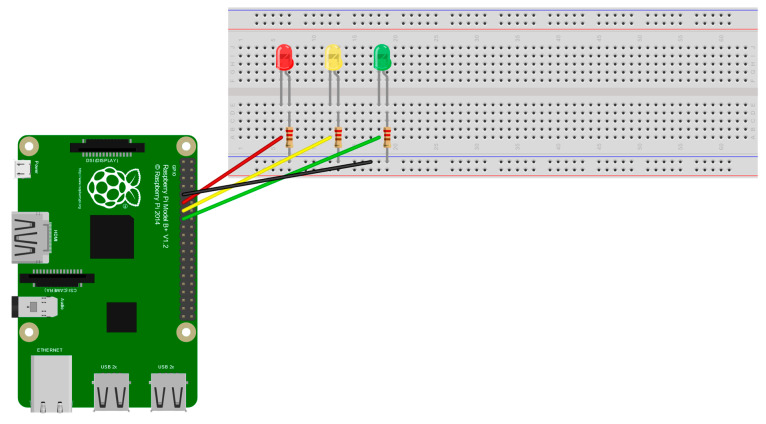
Proposed circuit to connect to the Raspberry Pi.

The circuit, in Figure 10, was used through a protoboard to perform light-on tests, according to the identified class. The Raspberry Pi sends a signal through its GPIO Board turning on the green LED in case the mask is placed correctly, yellow in case the mask is placed incorrectly, and red when the mask is not placed. The camera of the Raspberry Pi is placed at strategic points, according to the need to monitor in real-time and continuously detect people. 

## 4. Results and Discussion

The proposed model was tested in 30 experiments, in Table 2 it can be seen that the training that obtained the best results was number 18, in which 99.69% accuracy and 2.15% loss were obtained. These results compared to [18] are better on average, using the same datasets, in a similar way, Python libraries such as Keras, OpenCv, and Tensorflow were used.

The average obtained through the 30 trainings is of 99.60% with a standard deviation of 0.04%. Achieving a mean not so far from the best average obtained in experiment 18 and a very small standard deviation, so the separation between the average and the mean value is small.

**Figure 11:**
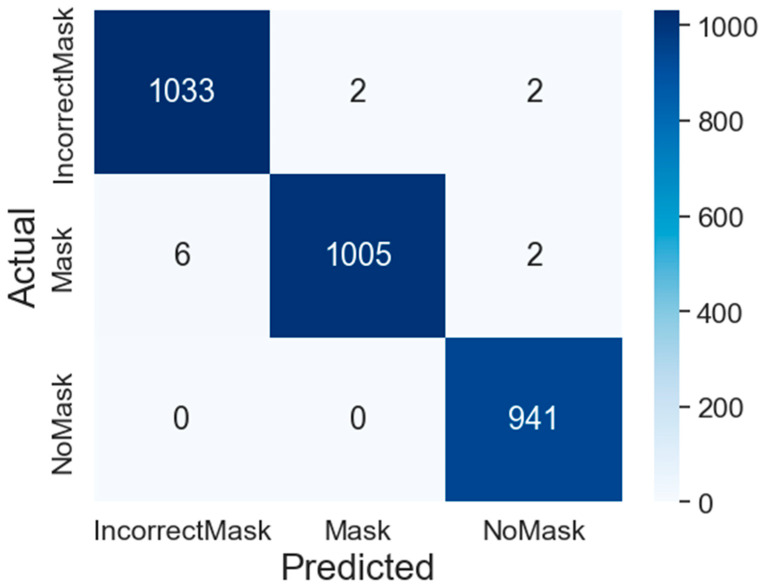
Confusion matrix of the best experiment.

The confusion matrix of the training with the best results is observed in Figure 11, where the heat map is shown with the evaluated images of the training percentage.

Of the 2991 images, we find that 2979 images were correctly classified in their respective classes, which in percentage it would be 99.60%, and is quite good according to the MaskedFace-Net dataset.

We classify different parts of the MaskedFace-Net to corroborate the efficacy of the proposed model, where the first part uses the first 15,000 images of the dataset, while part 2 the next 15,000. In the same way, part 3 evaluates the subsequent images, and part 4 uses the last images of the dataset.

As can be seen in Table 3, where the different parts of the dataset are evaluated, the best percentage for obvious reasons was obtained with Part 1, achieving 99.90% accuracy. This is because that part of the classification model of the use of face masks was trained, and although the percentage of accuracy is not higher in the other parts, this is still quite similar to obtaining percentages of high precision and low loss. This was achieved with the images of the dataset with which our model was not trained.

**Figure 12:**
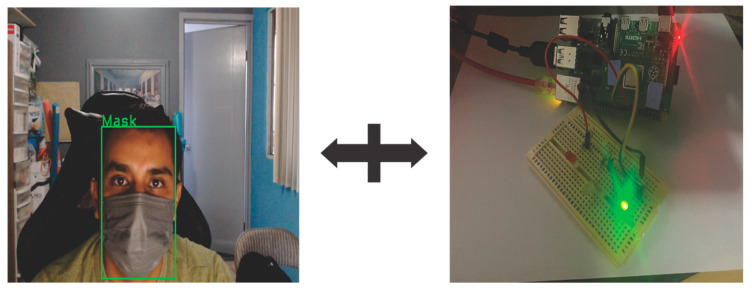
Example of the real-time system in Mask class.

Multiple tests were performed using a video camera to obtain the input images of the model so that it can be evaluated in real time. In Figure 12 the result of the classification of an image when a human is wearing the mask is shown.

In addition to classifying the class to which it belongs, at the same time it lights an LED according to the class, in this case the class is green color, because the mask is placed correctly. Through labeling, it is easier to detect humans who are not using the mask correctly, managing to detect if they belong to any of the three classes, which are Mask, NoMask, or IncorrectMask. In most cases, the results are quite satisfactory, managing to classify mask use correctly.

**Figure 13:**
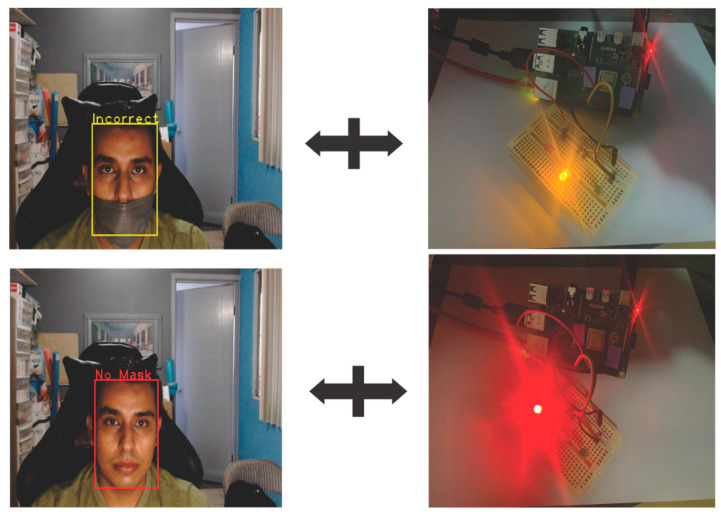
Example of the real-time system in IncorrectMask and NoMask class.

Figure 13 shows the classification of the remaining two classes, as well as the LED, which shows the color corresponding to the class, the yellow color for the IncorrectMask class, and the red color for the NoMask class. 

The Raspberry Pi 4 sends the signals to turn on the corresponding LED thanks to the GPIO Board and the programming made in Python to activate the appropriate pin and load the model for face detection and the model trained for the multiclass classification of the correct use of masks.

## 5. Conclusions

The proposed method is capable of classifying the correct use of multiclass face masks using a CNN model, in combination with computer vision. This is with the goal of avoiding transmitting the COVID-19 virus or any other virus that can be transmitted by air and this is achieved through real-time monitoring in strategic areas, by means of a Raspberry Pi 4, where it is possible to perform this identification to perform specific actions. 

The method will detect the face of a person and put a rectangular box labeled as Mask if the person is using a mask correctly, which happened when a mask is covering the nose, mouth, and chin. Otherwise, if the person is wearing the mask only covering the chin and mouth then the model will label it in a rectangular box as Incorrect, or if the person is not wearing any mask or if it is only on the chin then the label NoMask will show.

The model manages to classify the use of masks In three classes: NoMask, Mask, and IncorrectMask, and through the GPIO Board of the Raspberry Pi, sends signals to light green, yellow, or red LED, respectively, obtaining an accuracy percentage of 99.69%, evaluated with the MaskedFaceNet dataset.

The proposed system, therefore, will potentially help in decreasing the spread of the virus, helping people’s health systems. This solution could prevent restrictions from being breached in real time, improving the safety of people around us, and can be used in various areas such as schools, squares, and public or private places, among others.

## Figures and Tables

**Table 1:** Work-related to the classification of the appropriate use of face masks.

1st Author	Classification Model	Dataset	Classification Type	Software	Accuracy
Sethi [31]	CNN	MAFA *	Binary	PyTorch	98.2%
Deshmukh [19]	MobileNetV2	RFMD *, MaskedFaceNet	Multiple	-	99%
Bhattarai [26]	ResNet50	Kaggle [32], MaskedFaceNet	Multiple	OpenCV, Tensorflow, Keras	91%
Pham-Hoang-Nam [27]	ResNet50	Kaggle [32,33], MaskedFaceNET, MAFA *	Multiple	Tensorflow, Keras	94.59%
Yu [23]	YOLO-v4 Improved	RFMD *, MaskedFaceNet	Multiple	-	98.3%
Aydemir [34]	CNN	Manual, MaskedFaceNet	Multiple	MATLAB	99.75
Soto-Paredes [28]	ResNet-18	MaskedFaceNet, Kaggle	Multiple	PyTorch	99.05
Wang [25]	InceptionV2	RMFRD *, MAFA *, WIDER FACE, MaskedFaceNet	Multiple	OpenCV, MATLAB	91.1%
Rudraraju [21]	MobileNet	RMFRD *	Multiple	OpenCV, Keras	90%
Jones [18]	CNN	MaskedFaceNet	Multiple	Tensorflow, Keras	98.5%
The method proposed in this Paper	CNN + Preprocessing	MaskedFaceNet	Multiple	Tensorflow, Keras, OpenCV	99.69%

* MAFA (Masked Face), Masked Face Detection Dataset (MFDD), Real-world Masked Face Recognition Dataset (RMFRD), Simulated Masked Face Recognition Dataset (SMFRD), Simulated Facemask Dataset (SFMD), Real Facemask Dataset (RFMD).

**Table 2:** Experiments of the proposed CNN model.

Training	Accuracy	Loss	Training	Accuracy	Loss
1	0.9958	0.0378	16	0.9958	0.0250
2	0.9958	0.0494	17	0.9958	0.0325
3	0.9958	0.0454	18	0.9969	0.0215
4	0.9958	0.0283	19	0.9958	0.0388
5	0.9958	0.0669	20	0.9958	0.0613
6	0.9958	0.0752	21	0.9958	0.0578
7	0.9958	0.0621	22	0.9958	0.0599
8	0.9958	0.0675	23	0.9958	0.0510
9	0.9958	0.0338	24	0.9958	0.0319
10	0.9958	0.0534	25	0.9958	0.0541
11	0.9958	0.0256	26	0.9969	0.0285
12	0.9958	0.0571	27	0.9958	0.0467
13	0.9969	0.0335	28	0.9958	0.0638
14	0.9969	0.0436	29	0.9958	0.0705
15	0.9958	0.0443	30	0.9958	0.0268
			Average	0.9960	
			Standard deviation	0.0004	

**Table 3:** Evaluating the model with MaskedFace-Net parts.

Part	Accuracy	Loss
1	0.9990	0.0085
2	0.9975	0.0241
3	0.9980	0.0169
4	0.9963	0.0375

## Data Availability

Not applicable.
